# In-network multidisciplinary digital care improves outcomes in medicare advantage members with musculoskeletal diagnoses

**DOI:** 10.3389/fdgth.2026.1731986

**Published:** 2026-05-28

**Authors:** Austin George Cross, Usmaan Zunnu Rain, Eric Makhni, Peter Watson, Charles Bloom

**Affiliations:** 1 Department of Orthopaedic Surgery, Texas Tech University Health Sciences Center, Lubbock, TX, United States; 2 Department of Orthopaedic Surgery, Henry Ford Health System, Detroit, MI, United States; 3 Protera Health, Troy, MI, United States; 4 Populance, Henry Ford Health, Detroit, MI, United States; 5 Division of Hospital Medicine, Henry Ford Health, Detroit, MI, United States; 6 Health Alliance Plan of Michigan Inc, Detroit, MI, United States

**Keywords:** integrated practice unit, medicare, musculoskelatal conditions, patient report outcome measure, telehealth

## Abstract

**Introduction:**

Musculoskeletal (MSK) conditions are a major cost driver for Medicare Advantage (MA) plans. Digital health can improve access to preventive treatments like exercise therapy, but adoption has been challenging in older populations. This study evaluated the effectiveness of a novel, multidisciplinary virtual integrated practice unit (V-IPU) for MA members with MSK conditions.

**Methods:**

We retrospectively analyzed 308 consecutive MA beneficiaries completing a 12-week V-IPU. Clinical outcomes at baseline, 6, and 12 weeks included PROMIS Global-10 (physical and mental health), PROMIS Pain Interference (SF4a), PHQ-2, GAD-2. Engagement and satisfaction were also evaluated.

**Results:**

Participants improved in PROMIS physical health (+4.7), pain interference (–4.3), and mental health (+2.7) domains, with 93.8% achieving minimal clinically important difference (MCID). Depression and anxiety scores improved by 35.7% and 24.6%, respectively. Engagement and satisfaction of the V-IPU was high.

**Conclusion:**

The novel, physician-led V-IPU significantly improved function, pain and mental health in MA members with MSK conditions. Broader use may help improve outcomes and reduce cost for risk-bearing organizations.

## Introduction

Musculoskeletal (MSK) conditions are consistently among the top cost drivers for health insurance plans (1). These costs are driven by high disease burden (one in two American adults have back, neck, or joint pain) and fee-for-service payment infrastructure that prioritizes quantity over quality of services delivered. Whereas costs temporarily declined during the height of the COVID-19 pandemic, utilization of MSK care has rebounded and continued to climb since then.

In reaction to these climbing costs, health plans and self-insured employers have turned to digital health technologies with the hopes of increasing access to preventative treatment, especially through exercise therapy. These programs consist of self-guided exercise programs, often accompanied with wearable sensors or motion capture technology (2), and have been widely adopted in commercial populations, especially with self-insured employers (3, 4). However, adoption has been slower in Medicare Advantage (MA) populations due to inherent challenges with technology adoption, higher MSK medical complexity, and presence of multiple comorbidities in this demographic.

The purpose of this study was to measure the clinical and engagement outcomes of a novel, physician-led, multidisciplinary digital solution in MA members with MSK conditions. This virtual integrated practice unit (“V-IPU”) was modeled on the multidisciplinary delivery program popularized by Lee and Porter (5). We hypothesize that the increased clinical rigor, care team delivery model, and emphasis on live, synchronous telehealth care of the V-IPU will result in significant improvement in clinical outcomes of participating patients. We additionally hypothesize that the program will demonstrate strong engagement (as measured by program completion rate and weekly participation) and experience/satisfaction scores.

## Methods

This study received Institutional Review Board approval and consisted of a retrospective review of prospectively collected data of 308 Medicare Advantage members that participated in the V-IPU (Protera Health, Troy, MI). All members were from a single health insurance plan (Health Alliance Plan, Troy, MI).

**Figure 1:**
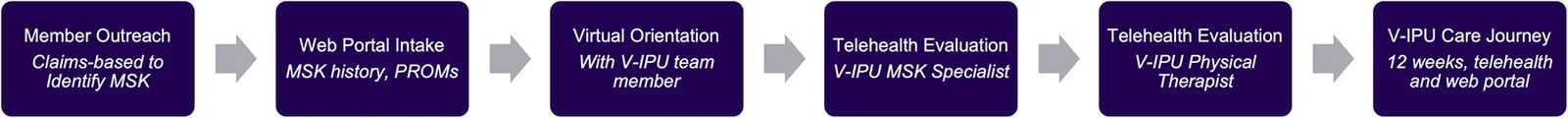
Flow diagram demonstrating patient onboarding process.

The study cohort consisted of 308 consecutive health plan members that completed the V-IPU program. Members were identified for enrollment if they had a health insurance claim with an MSK diagnosis corresponding to back, neck, hip, knee, or shoulder pain within the preceding 12 months. Outreach was performed telephonically. Interested members then met with a member of the V-IPU care team for a virtual, 1:1 orientation. This visit served to confirm that the member had active symptoms that may respond well to participation in the program. If the member had concerns that required surgical evaluation, the care team member provided concierge assistance to an in-network surgeon or specialist and facilitated an appointment. A detailed onboarding flow can be seen in Figure 1. Following this orientation, each member received a package consisting of a welcome card, set of exercise bands, and a cellular phone holder to facilitate hands-free telehealth encounters.

Upon enrolling into the 12-week program, each patient underwent an initial 1:1 telehealth evaluation (20–30 min) by an MSK clinician (orthopedic surgeon, primary care sports medicine physician, or specialty advanced practice provider). This encounter consisted of a detailed, and relevant, MSK history along with a virtual physical examination of the affected joint/body part. The provider could order imaging if desired or prescribe non-narcotic pain medication as well. If the provider felt an in-person consultation with an MSK specialist was warranted, a member of the V-IPU care team would facilitate such a referral (as described above).

The patient then underwent a subsequent, 1:1, synchronous telehealth evaluation and treatment session (60 min) by a licensed Doctor of Physical Therapy (DPT). This encounter included a structured assessment of pain, function, range of motion, and movement patterns, along with review of relevant medical history and establishment of an individualized treatment plan. The member also began the exercise program under virtual supervision of the physical therapist during that encounter. Following this session, the member received weekly or biweekly telehealth sessions from the V-IPU care team, consisting of physical therapists along with physicians, registered dietitians, and health coaches. Each subsequent encounter was 1:1 between the participant and a member of the care team at a cadence recommended by the physical therapist and/or medical provider. The care team participated in regular synchronous and asynchronous “huddles”, led by the clinical provider, to determine need for any clinical escalation to a V-IPU orthopedic surgeon or primary care sports medicine physician, as appropriate.

Throughout the program, “red flag” signs were assessed to ensure appropriateness of program participation, with referral to appropriate in-person specialists or emergency settings if warranted. Program participants were educated about common red flag signs through their video education, and V-IPU providers were similarly trained on assessing and diagnosing such signs during the course of clinical evaluation. These red flag signs included, but were not limited to, unexplained weight loss/gain, bowel/bladder incontinence, progressive motor or sensory dysfunction, signs of infection, and acute medical/psychiatric events.

### V-IPU web portal

Each participant received access to a web portal that contained an individualized education and exercise program along with a secure text message interface for communication with the V-IPU care team. The PT assigned weekly exercise programs that could be accessed and logged from the web portal. The care team also assigned educational videos covering various topics relevant to MSK care, including nutrition, mental health, pain coping, and condition-based information. The V-IPU care team was able to prescribe non-narcotic pain medication or order radiographic (or advanced) imaging as clinically necessary throughout the program. If patients required triage to in-person specialist providers, the V-IPU care team facilitated the appointment to a qualified in-network provider.

### Clinical outcomes assessment and study objectives

Each patient underwent clinical outcomes assessment at enrollment, as well as at 6-weeks and 12-weeks following enrollment. Clinical outcomes consisted of:

- Patient reported outcome measures (PROMs) – National Institutes of Health (NIH) Patient-Reported Outcomes Measurement Information System (PROMIS) Global 10 and Pain Interference (SF4a), GAD-2 anxiety screen, PHQ-2 depression screen
- Patient activation, as measured by a *de novo* assessment and measuring the understanding, skills, and confidence of making positive lifestyle choices
- Social determinants of health (SDOH) measuring access to nutrition and transportation
- Patient demographics: date of birth/age, height/weight (to calculate Body Mass Index, or BMI), and sex

For this clinical study, the primary outcome measures were change in PROM scores for physical health (from the PROMIS-10), mental health (subscale from PROMIS-10), impact of pain on quality of life (PROMIS Pain Interference Short Form 4a), and scores on the depression (PHQ-2) and anxiety (GAD-2) screens. For detecting minimum clinically important difference (MCID), a threshold of 2.5 points was used for PROMIS Physical Health and Pain Interference (6). These two domains were included in MCID analysis due to the nature of the V-IPU as a treatment for MSK conditions and goal of improving physical and/or pain health.

Additionally, program engagement was measured, consisting of average weekly interactions with the digital program (care team appointments and/or web portal logins) and educational videos consumed. Finally, patient experience scores were measured with regards to average satisfaction rating (on a scale from 1 to 10, with 10 being the most likely to refer the program to a friend or family member) and net promoter score (or NPS). NPS is based on the participant's response to the question of “How likely is it that you would recommend our company to a friend or colleague?”. Participants choose a numerical value 0–10, with 0 representing “not at all likely” and 10 representing “extremely likely”. Those who respond with values 0–6 are considered “detractors”, 7-8 being “passive”, and finally those who responded 9-10 are considered “promoters”. NPS was calculated with the following formula: NPS=% of promoters - % of detractors (7).

## Results

Our study included a total of 308 consecutive MA patients that completed the V-IPU. There were 227 females and 79 males (2 participants did not enter gender data), with the average age of 69 years. Full demographic information can be visualized in Table 1. The most common injury location was the back (39.6%), followed by the knee (18.2%) and shoulder (12%).

**Table 1:** Patient demographical information.

Demographical Variable	*N* = 308	Percentage (%)
Age		
<50	2	0.65
50–60	23	7.5
61–70	134	43.5
71–80	142	46.1
81–90	7	2.3
>90	0	0
Sex		
Male	79	25.6
Female	227	73.7
No response	2	0.65
Race		
Black/African American	68	28.2
White	155	64.3
Hispanic	0	0
Asain	2	0.83
Other	4	1.7
Mixed	9	3.7
No response	67	
BMI		
<18.5	1	1
18.5–24.9	53	17.7
25–30	86	28.8
30–35	74	24.7
35–40	46	15.4
>40	39	13
No response	8	
Chief complaint		
Knee	56	18.2
Arm/elbow	2	0.6
Back	122	39.6
Hand/wrist	8	2.6
Hip/thigh	28	9.1
Neck	29	9.4
Shoulder	37	12
Other	17	5.5
Unknown	1	0.3

**Figure 2:**
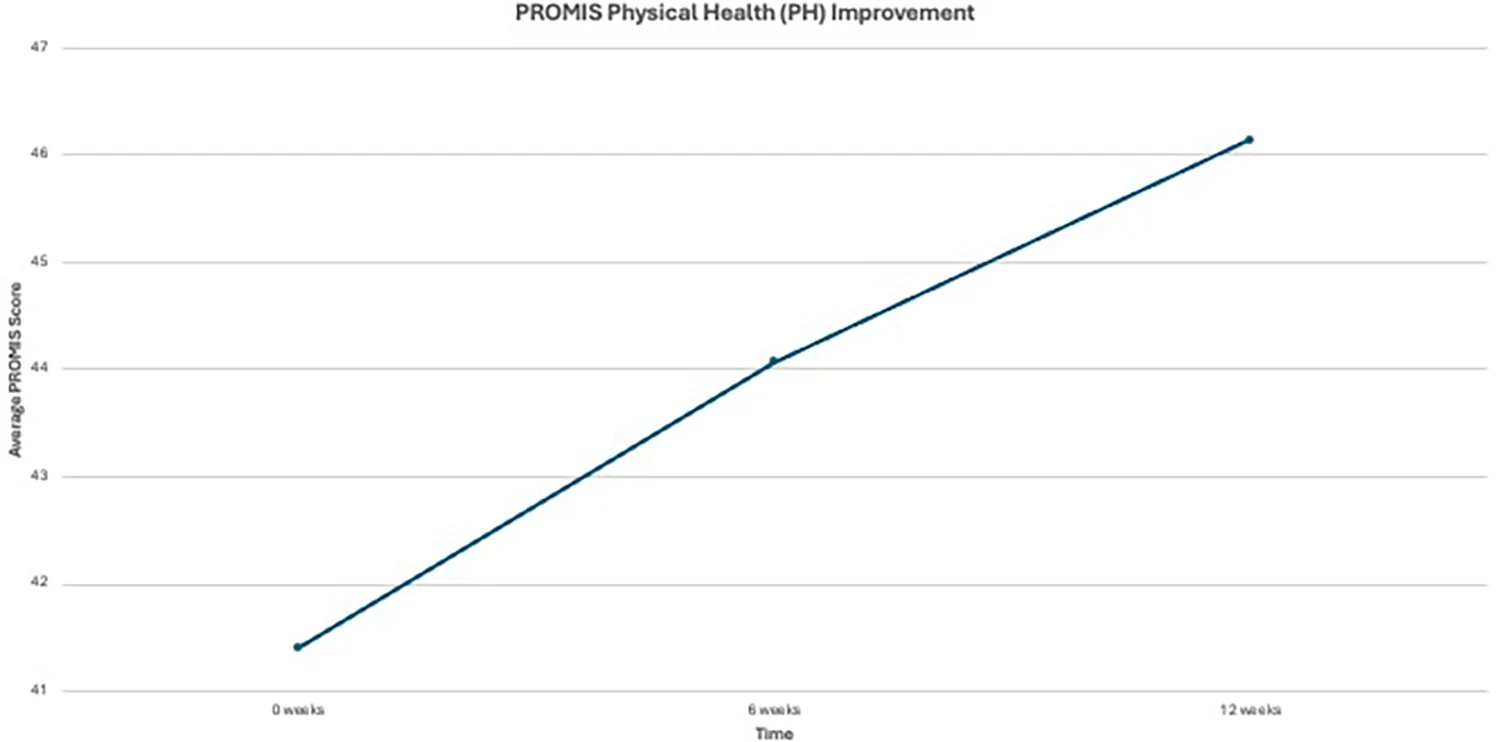
Average PROMIS physical health improvement over the 12-week time period.

**Figure 3:**
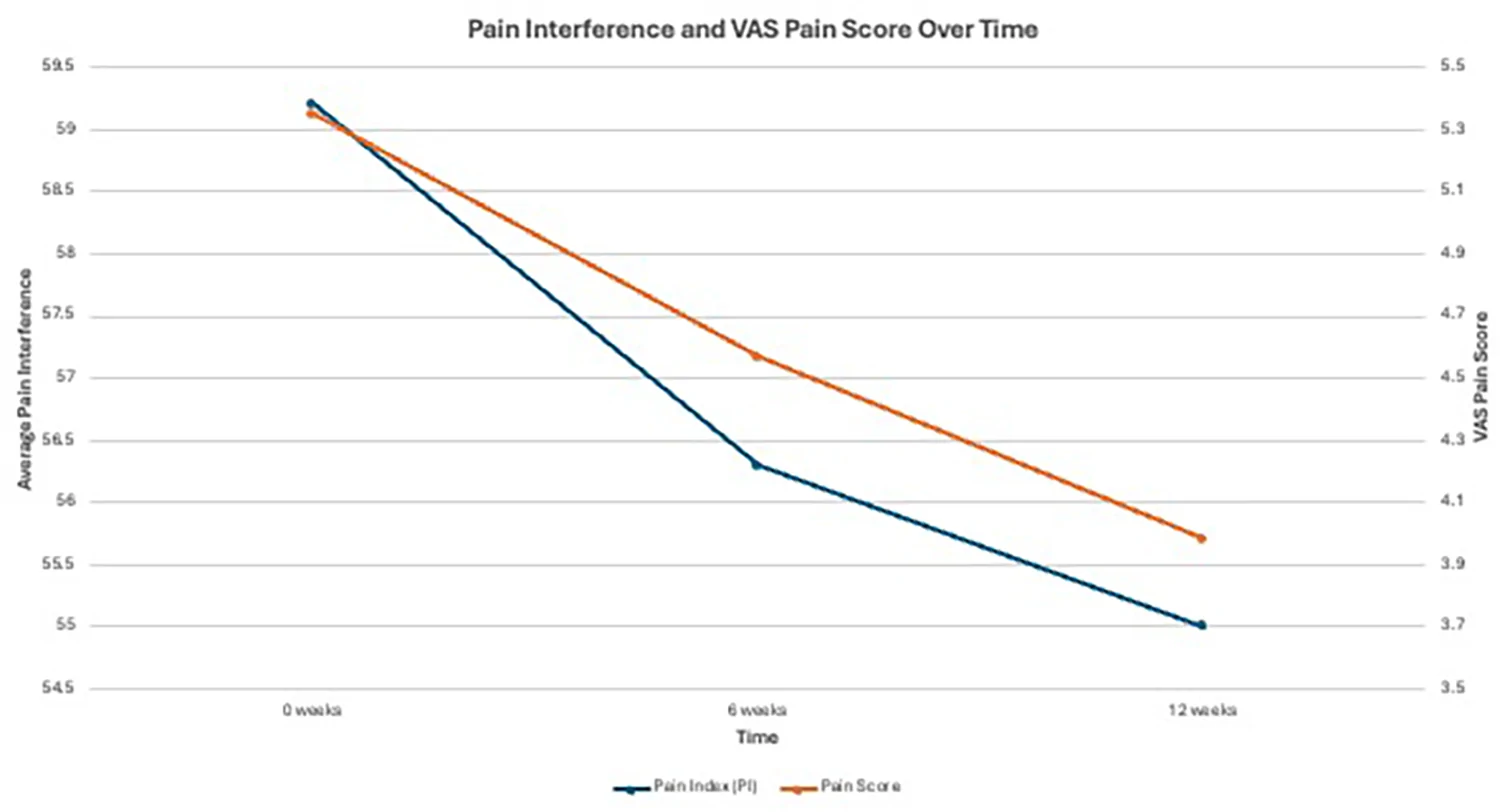
Average PROMIS pain interference and VAS pain improvement over the 12-week time period.

**Figure 4:**
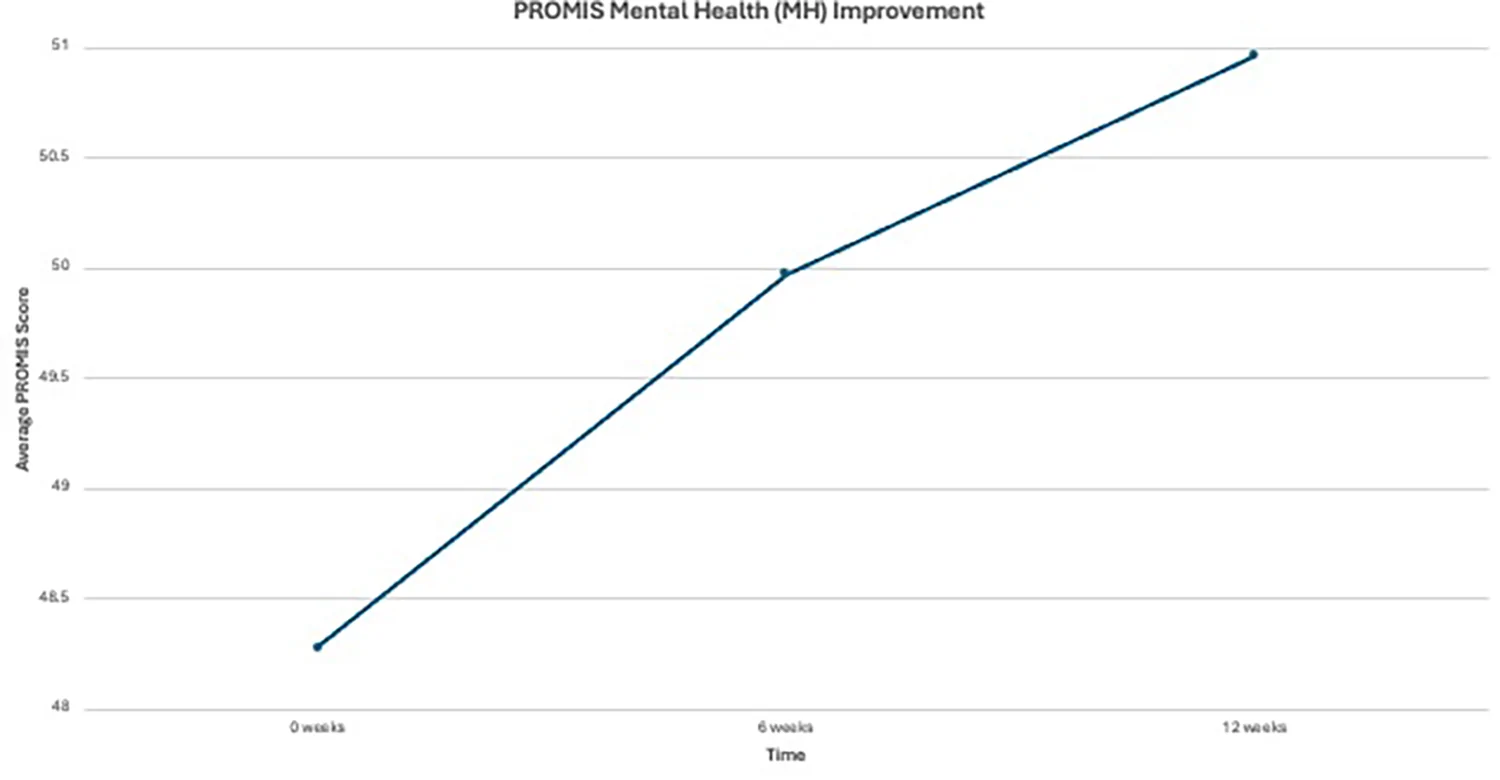
Average PROMIS mental health improvement over the 12-week time period.

**Figure 5:**
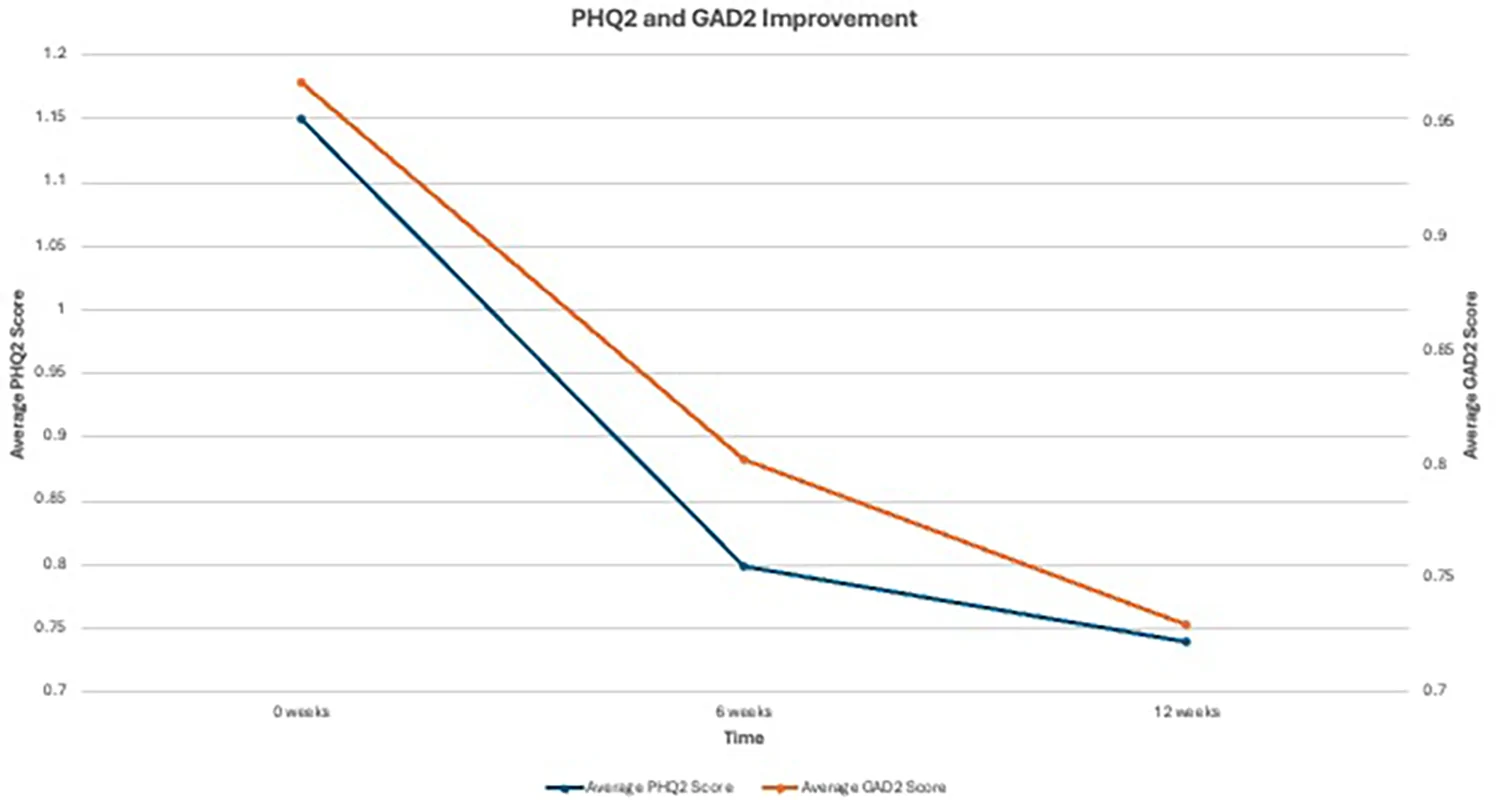
Average improvement of the patent-health questionnaire-2 (PHQ-2) and general anxiety disorder 2-item (GAD-2) questionnaires.

**Figure 6:**
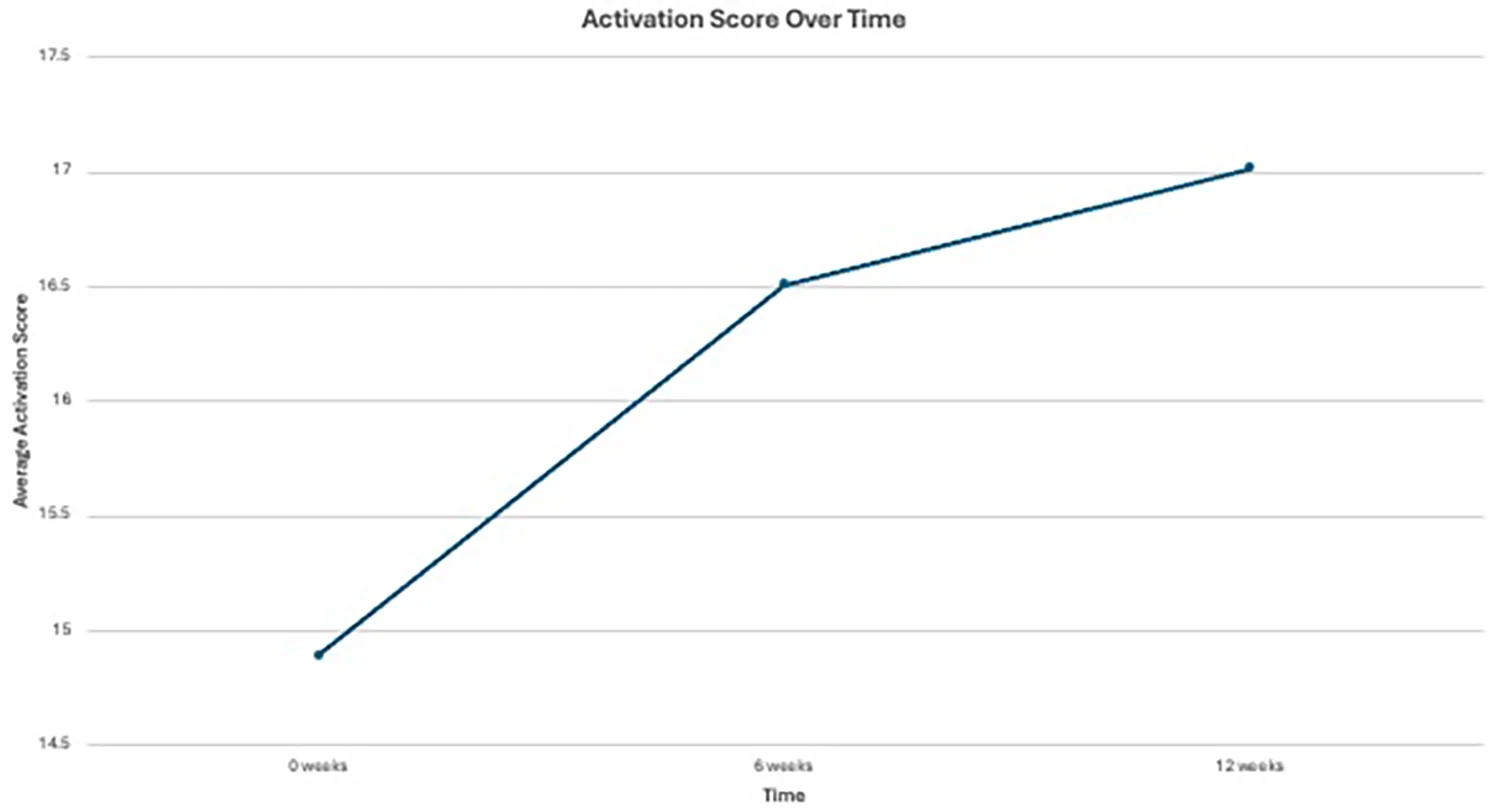
Average participant activation score over the 12-week study duration.

With regards to PROM scores, there was an improvement of 4.7 points on the PROMIS-10 physical health component (baseline score=41.4; follow-up score=46.1), which can be visualized in Figure 2. There was a 4.3 point improvement in pain interference (baseline score=59.3; follow-up score=55.0), and 1.4 improvement in the visual analog pain scale (VAS) (baseline score=5.3; follow-up score=3.9) which can be seen in Figure 3. For mental health, the PROMIS-10 mental health component improved a total of 2.7 points (from 48.3 to 51.0). The improvement for the PROMIS-MH can be visualized in Figure 4. There was a 35.7% (baseline 1.2, follow-up score 0.7) and 24.6% (baseline 0.9, follow-up score 0.7) improvements in PHQ-2 Depression Screen and GAD-2 Anxiety Screen scores, respectively (Figure 5). Finally, patient activation scores improved by approximately 14% (baseline 14.9 points, follow-up score 17.0), which can be visualized in Figure 6.

Of the 308 members, 93.8% (*n* = 289) achieved minimum clinically important difference (MCID) for either physical health or pain interference improvement (MCID threshold set at 2.5 points for PROMIS PH and PROMIS PI).

When considering engagement with the V-IPU, there was an average of 52.4 active days (web portal logins or care team telehealth appointments) per week during the 12-week care journey per participating health plan member. On average, each participant completed the following number of telehealth encounters: 1.2 telehealth medical/clinical, 7.4 physical therapy, and 4.2 with the health coach. Within the web portal, there were 27.4 educational videos and articles consumed per participant. The average satisfaction rating was 9.4 out of 10, corresponding to a net promoter score (NPS) of 83.

## Discussion

In Medicare Advantage members with musculoskeletal conditions, the V-IPU was effective in improving clinical outcomes for physical, mental, and pain health, with an overwhelming majority of patients improving to a clinically meaningful degree. Additionally, this study also demonstrated strong engagement and satisfaction with the digital program.

The implications of this study are important when considering the pressing need to further adopt value-based care delivery in today's costly healthcare landscape. Increasingly, health insurance plans, self-insured employers, and even provider organizations are taking financial risk for large populations (5, 8, 9). This shift has resulted in a priority for cost-conscious care delivery and emphasis on avoidance of unnecessary procedures and interventions.

Unfortunately, musculoskeletal (MSK) care is highly variable with regards to quality and appropriateness of treatment decision-making (10–14). Many health insurance plans have attempted to curb unnecessary procedures and interventions through use of “downstream” utilization management, in which third-party vendors enforce prior authorization in order to proceed with planned treatment. This practice has demonstrated not only questionable efficacy (15, 16) but also significant friction between the health insurance plan and its participating members and physicians. As a result, there are active efforts from both regulatory (17) and grassroots (18) movements, to curb the use of this controversial cost-containment strategy.

One promising strategy for cost containment is through “upstream” clinical interventions that are designed to improve patient health outcomes, thereby reducing the need for costly procedures and surgeries. The most notable such strategy is that of integrated practice units (IPUs), which were first described by Porter and Lee in 2013 (5). In an IPU delivery model, patients are managed holistically for a given condition (e.g., knee osteoarthritis, low back pain, etc.). Additional features of the IPU model include strict measurement of clinical outcomes (through PROMs) and multidisciplinary care teams that provide longitudinal care. Unfortunately, despite the promise of such models, they are incredibly challenging to scale in traditional “brick-and-mortar” clinical settings due to numerous administrative, logistical, technological, and financial challenges (19, 20) for both patient and clinicians.

In this study, a V-IPU was used to decrease variability of care and therefore optimize clinical and engagement outcomes. In particular, the V-IPU in this study utilized care pathways that were standardized. From a clinical perspective, each health plan member received a similar cadence of clinical, physical therapy, and health coach care. There were structured care team huddles to review new health plan participants and identify any that may benefit from escalation to a V-IPU or in-person MSK specialist. Each participant also received standardized – yet individualized – educational video programming regarding that participant's MSK condition, red flag signs, and pertinent content to nutrition, weight loss, value-based treatment modalities, pain coping, and mental health wellness. The V-IPU also conducted robust, regimented clinical outocmes assessments at intake, mid-point, and conclusion of the 12-week treatment journey.

This study represents the largest digital MSK intervention with physician-led care in the Medicare Advantage population. We identified one published study that examined outcomes following digital health intervention in Medicare Advantage members. In this study (21), 100 patients with MSK conditions underwent the digital intervention. However, follow-up physical health (also from the PROMIS-10) was available in only 57 patients, with an average improvement of 5 points. Our study reported similar physical function improvement but with a follow-up cohort of over 300 patients. Additionally, our study reported on pain interference scores, which are critical measures that quantify the impact of pain on quality of life. Traditional visual analog score pain reporting (i.e., on scale from 1 to 10) has been found to be inferior to pain interference (22, 23).

This study was not without limitations. First, patients came from a singular health insurance plan; therefore, the results may not be generalizable to the broader Medicare Advantage population. Secondly, as this was a study on clinical and engagement outcomes following digital health treatment, there were no correlations or measurements made for functional outcomes (e.g., strength), imaging findings, or cost of care/utilization metrics. Nor was there an effective way to participate for health plan members that lacked access to these technologies. However, given the requirement of only a web-enabled device or smartphone, and ability to participate with assistance from a family member, the digital program was able to maximize access to health plan members. Third, the follow-up time period was set at 12 weeks. While this time range is adequate for completing a thorough nonoperative treatment (24–26), a longer follow-up time would be helpful, as would analysis on achievement of additional cut points for indicating clinical improvement (in addition to the 2.5 point threshold). Fourth, the care team was defined as including a medical provider, physical therapist, and health coach. It did not include mental health or social work professionals, which may have limited some ability to manage complex MSK cases. A fifth limitation is the lack of a control group. While a control group would have been helpful for studying efficacy of the intervention, it was not possible to construct due to the lack of clinical outcome measurements associated with traditional, in-person health care. An additional limitation is related to the ability to measure patient satisfaction, which was done via “Net Promoter Score” and 1–10 rating. Such a global rating may be limited in discerning granular assessments of satisfaction for different parts of the treatment episode. Finally, exact counts of referral to in-person specialists were not recorded as part of this study.

## Conclusion

In conclusion, the virtual IPU intervention resulted in meaningful clinical improvements for physical function, mental health, and pain interference in Medicare Advantage members with musculoskeletal conditions. Given the high-cost burden of MSK conditions in this population, expanded use of this intervention has the potential to not only improve health outcomes but also decrease expenditures for risk bearing organizations.

## Data Availability

The raw data supporting the conclusions of this article will be made available by the authors, without undue reservation.
