# Correction: In-network multidisciplinary digital care improves outcomes in medicare advantage members with musculoskeletal diagnoses

**DOI:** 10.3389/fdgth.2026.1898586

**Published:** 2026-08-19

**Authors:** Austin George Cross, Usmaan Zunnu Rain, Eric Makhni, Peter Watson, Charles Bloom

**Affiliations:** 1 Department of Orthopaedic Surgery, Texas Tech University Health Sciences Center, Lubbock, TX, United States; 2 Department of Orthopaedic Surgery, Henry Ford Health System, Detroit, MI, United States; 3 Protera Health, Troy, MI, United States; 4 Populance, Henry Ford Health, Detroit, MI, United States; 5 Division of Hospital Medicine, Henry Ford Health, Detroit, MI, United States; 6 Health Alliance Plan of Michigan Inc, Detroit, MI, United States

**Keywords:** integrated practice unit, medicare, musculoskelatal conditions, patient report outcome measure, telehealth

There was a mistake in Table 1 as published. Under the “Chief Complaint” sub-heading, the table lists “Foot/ankle” with a *N* of 122 and Percentage of 39.6. “Foot/ankle” is incorrect and should be changed to “Back”. The corrected Table 1 appears below.

**Table 1:** Patient demographical information.

Demographical Variable	*N* = 308	Percentage (%)
Age		
<50	2	0.65
50–60	23	7.5
61–70	134	43.5
71–80	142	46.1
81–90	7	2.3
>90	0	0
Sex		
Male	79	25.6
Female	227	73.7
No response	2	0.65
Race		
Black/African American	68	28.2
White	155	64.3
Hispanic	0	0
Asain	2	0.83
Other	4	1.7
Mixed	9	3.7
No response	67	
BMI		
<18.5	1	1
18.5–24.9	53	17.7
25–30	86	28.8
30–35	74	24.7
35–40	46	15.4
>40	39	13
No response	8	
Chief Complaint		
Knee	56	18.2
Arm/Elbow	2	0.6
Back	122	39.6
Hand/Wrist	8	2.6
Hip/Thigh	28	9.1
Neck	29	9.4
Shoulder	37	12
Other	17	5.5
Unknown	1	0.3

The original version of this article has been updated.

